# Re-modeling of foliar membrane lipids in a seagrass allows for growth in phosphorus-deplete conditions

**DOI:** 10.1371/journal.pone.0218690

**Published:** 2019-11-27

**Authors:** Jeremy P. Koelmel, Justin E. Campbell, Joy Guingab-Cagmat, Laurel Meke, Timothy J. Garrett, Ulrich Stingl

**Affiliations:** 1 University of Florida, Department of Pathology, Immunology, and Laboratory Medicine, College of Medicine, Gainesville, Florida, United States of America; 2 Florida International University, Department of Biological Sciences, Institute of Water and Environment, North Miami, FL, United States of America; 3 University of Florida, UF/IFAS Fort Lauderdale Research and Education Center, Department of Microbiology & Cell Science, Davie, Florida, United States of America; Università della Calabria, ITALY

## Abstract

In this study, we used liquid chromatography high-resolution tandem mass spectrometry to analyze the lipidome of turtlegrass (*Thalassia testudinum*) leaves with either extremely high phosphorus content or extremely low phosphorus content. Most species of phospholipids were significantly down-regulated in phosphorus-deplete leaves, whereas diacylglyceryltrimethylhomoserine (DGTS), triglycerides (TG), galactolipid digalactosyldiacylglycerol (DGDG), certain species of glucuronosyldiacylglycerols (GlcADG), and certain species of sulfoquinovosyl diacylglycerol (SQDG) were significantly upregulated, accounting for the change in phosphorus content, as well as structural differences in the leaves of plants growing across regions of varying elemental availability. These data suggest that seagrasses are able to modify the phosphorus content in leaf membranes dependent upon environmental availability.

## Introduction

Seagrasses are a widely distributed group of marine plants that provide a range of ecological services to coastal habitats around the world. However, due to a variety of natural and anthropogenic stressors, many seagrass beds are declining globally[1]. For over 30 years, it has been known that many seagrass species can display shifts in foliar phosphorus (P) content in response to environmental availability[2,3], an adaptation that allows for growth in a wide range of habitats of varying nutrient regimes. In *Thalassia testudinum* (turtlegrass), a dominant species in South Florida[4], elemental C:P ratios can differ by nearly 8-fold, from around 400 to 3000, often dependent upon environmental P availability[3,5,6]. *Thalassia testudinum* is distributed along the western Atlantic from Florida, USA to Venezuela, throughout the Gulf of Mexico and the Caribbean Sea[7]. *Thalassia hemprichii*, the other species in this genus, is also widely distributed in the coastal waters of the Indian Ocean and the western Pacific[8]. While the morphology of turtlegrass leaves and canopy structure changes with decreased P content, areal production rates can remain relatively high[6], indicating metabolically active plants. Changes in C:P ratios and P content of turtlegrass occur along natural P gradients[4,6,9], but can also be induced by fertilization experiments in P-depleted habitats[5,10]. The exact cellular mechanisms on how turtlegrass lowers its P content are mostly unknown. In this study, we used liquid chromatography high-resolution tandem mass spectrometry (LC-HRMS/MS) to analyze the lipidome of turtlegrass leaves that contained either a high percentage of P or a low percentage of P as a result of a fertilization experiment.

## Materials and methods

### Sample collection

In 2014, samples of *Thalassia testudinum* leaf tissue were collected in Key Largo, Florida, as part long-term fertilization experiment (see Campbell et al 2018 for full details)[5]. In brief, 60 replicate 0.25 m^2^ plots were established in a shallow (1 m deep) *T*. *testudinum* meadow in Largo Sound (25° 7.58’ N, 80° 24.29’ W). Thirty of these plots were haphazardly selected to receive amendments of slow release fertilizer. Nutrient enriched plots received 350 g of slow release Osmocote fertilizer (NPK 14-14-14) enclosed in fiberglass mesh bags. Each bag was attached to a PVC post positioned in the center of the plot and was replaced every 4 weeks to ensure consistent nutrient delivery. After 14 weeks, 3–4 separate shoots were harvested from each plot and transported to the laboratory on ice. Shoots from each plot were pooled, rinsed in deionized water, scraped free of epiphytic growth, and dried to a constant weight at 60°C. All samples were then pulverized, homogenized and stored in boroscillicate glass vials. Nitrogen content (% dry mass) was measured via CHN analysis (Fisons NA1500 elemental analyzer). Phosphorus (P) content (% dry mass) was measured colorimetrically after a dry-oxidation, acid hydrolysis extraction procedure[11]. Plots displaying the highest and lowest P content were then selected for further lipidome analysis. Elemental C, N, P contents of leaves of selected plots are shown in Table 1.

**Table 1:** Elemental composition of samples.

Sample ID	Plot	%N	%C	%P	C:N ratio	C:P ratio	N:P ratio	Limitation index
**KL-15**	**15**	**2.92**	**40.19**	**0.53**	**16.05**	**195.05**	**12.15**	**17.85**
**KL-45**	**45**	**2.95**	**35.79**	**0.44**	**14.16**	**209.70**	**14.80**	**15.20**
**KL-8**	**8**	**3.07**	**38.84**	**0.44**	**14.75**	**226.45**	**15.35**	**14.65**
**KL-20**	**20**	**2.95**	**40.41**	**0.43**	**15.96**	**243.71**	**15.27**	**14.73**
**KL-37**	**37**	**3.26**	**41.33**	**0.42**	**14.79**	**252.83**	**17.09**	**12.91**
**KL-10**	**10**	**2.98**	**41.08**	**0.41**	**16.09**	**258.68**	**16.08**	**13.92**
**KL-32**	**32**	**2.85**	**41.44**	**0.09**	**16.95**	**1154.33**	**68.12**	**38.12**
**KL-19**	**19**	**2.28**	**38.25**	**0.08**	**19.61**	**1177.24**	**60.04**	**30.04**
**KL-31**	**31**	**2.48**	**39.83**	**0.08**	**18.74**	**1234.59**	**65.90**	**35.90**
**KL-54**	**54**	**3.03**	**42.36**	**0.09**	**16.31**	**1281.66**	**78.59**	**48.59**
**KL-28**	**28**	**2.24**	**38.30**	**0.08**	**19.94**	**1293.30**	**64.85**	**34.85**
**KL-22**	**22**	**2.27**	**41.40**	**0.08**	**21.25**	**1343.90**	**63.25**	**33.25**

Plot number refers to plots described in Campbell et al 2018. Limitation index describes the relative divergence from the ideal N:P ratio for *Thalassia testudinum* (near 30:1). Orange: Plants with high P content, blue: plants with low P content.

### Sample preparation

Samples were extracted using the folch extraction procedure[12]. Briefly, 25 mg of seagrass was weighed and 10 μL of 10x diluted internal standard mixture (stock solution of 50 ppm, w:v) was added. Internal standards were purchased from Avanti Polar Lipids, inc. (Alabaster, AL, USA) and consisted of: LPC (17:0), PC (17:0/17:0), PG (14:0/14:0), PE (15:0/15:0), PS (14:0/14:0), PI (8:0/8:0), SM (d18:1/17:0), Cer (d18:1/17:0), DG (14:0/14:0), CL (15:0_15:0_15:0_16:1), Sphingosine (d17:1), PAzePC, Glucosyl (β) Cer (d18:1/12:0), BMP (14:0/14:0) (S,R), and LSM (d17:1), except for TG (15:0/15:0/15:0), which was obtain from Nu-Chek (Elysian, MN, USA). Samples were extracted using 1:2:4 water:methanol:chloroform (v:v:v), and the organic phase was collected, dried down, and reconstituted in 75 μL of isopropanol plus 1 μL of injection standard mixture (100 ppm, w:v). Injection standards were purchased from Avanti Polar Lipids, Inc. (Alabaster, AL, USA) and consisted of: LPC (19:0), PC (19:0/19:0), PG (17:0/17:0), PE (17:0/17:0), PS (17:0/17:0), and TG (17:0/17:0/17:0). Extraction blanks (without internal standard), neat quality controls (QCs, blanks with internal standards), solvent blanks, and Red Cross plasma for QC purposes were also prepared.

### Data acquisition

Data was acquired using high-performance liquid chromatography high-resolution tandem mass spectrometry (LC-HRMS/MS). Chromatographic separation was achieved using reverse phase chromatography (Dionex Ultimate 3000 RS UHLPC system, Thermo Scientific, San Jose, CA, USA) with a Waters Acquity C18 BEH column maintained at 30°C (2.1 × 100 mm, 1.7 μm particle size, Waters, Milford, MA, US). The gradient (S1 Table) consisted of solvent A (60:40 acetonitrile:water) and solvent B (90:8:2 isopropanol:acetonitrile:water), both with 10 mM ammonium formate and 0.1% formic acid. The flow rate was 500 μL/min. Ammonium formate is not only needed for separation, but also for ionization of neutral lipids as [M+NH_4_]^+^ in electrospray ionization.

For acquiring mass spectra and MS/MS, a Q-Exactive orbitrap (Thermo Scientific, San Jose, CA) was used. Mass spectral parameters are shown in S2 and S3 Tables. The sequence consisted of three blanks followed by a neat QC, and one blank and QCs inserted between every 10 samples. Data was acquired for six low P containing seagrass samples and six high P containing seagrass samples injected at 2 μL in positive ion mode, and 4 μL in negative ion mode. Both data-dependent (ddMS^2^-top10) and all-ion fragmentation (AIF) data were obtained on two samples per group for identification purposes. In addition, full-scan data was acquire for all 12 samples without MS/MS for comparing lipid intensities across groups.

### Data analysis

LipidMatch Flow was used for file conversion, peak picking (implementing MZMine 2[13]), blank filtration[14,15], lipid annotation[16], and combining positive and negative datasets. LipidMatch Flow software and tutorials (including video tutorials) can be found at <http://secim.ufl.edu/secim-tools/>. In addition to LipidMatch annotation[16], MS-DIAL[17] annotations were appended to the feature table obtained from LipidMatch Flow using an in-house R script[18]. MS-DIAL was only used to identify lipids using data-dependent analysis while LipidMatch was used to annotate ions using both all-ion fragmentation (AIF) data and data-dependent analysis.

### Statistics

Multivariate statistical analysis was performed using Metaboanalyst 3[19]. Raw intensity values were normalized by sum, log transformed, and mean centered. Principal component analysis (PCA) was performed on the resulting normalized lipid values, with samples color coded by low P and control. For univariate statistics a two-tailed heteroscedastic t-tests was performed on low P versus control samples (peak areas). To account for multiple comparison errors, the Benjamini–Hochberg method[20] was used to obtain false discovery rate (FDR) corrected p-values. Features with lipid annotations and an FDR corrected p-value less than 0.05 were considered significant. To determine trends across lipid classes, a Fisher's exact test was performed using an in-house R[18] script. For the Fisher's exact test, features were considered upregulated with a lipid peak area fold change greater than 1.5 and downregulated with a fold change less than 0.67. In either case, features were only included if the FDR corrected p-value was less than 0.2. Lipid features and their respective classes according to this inclusion criteria were highlighted in a volcano plot color coded by lipid class. Fisher's exact test was used to calculate p-values based on whether lipids of a certain class tended to be more significantly upregulated or downregulated compared to lipids across all classes. Therefore, the lipid classes with the most significant change between low P and controls was determined.

## Results and discussion

**Fig 1:**
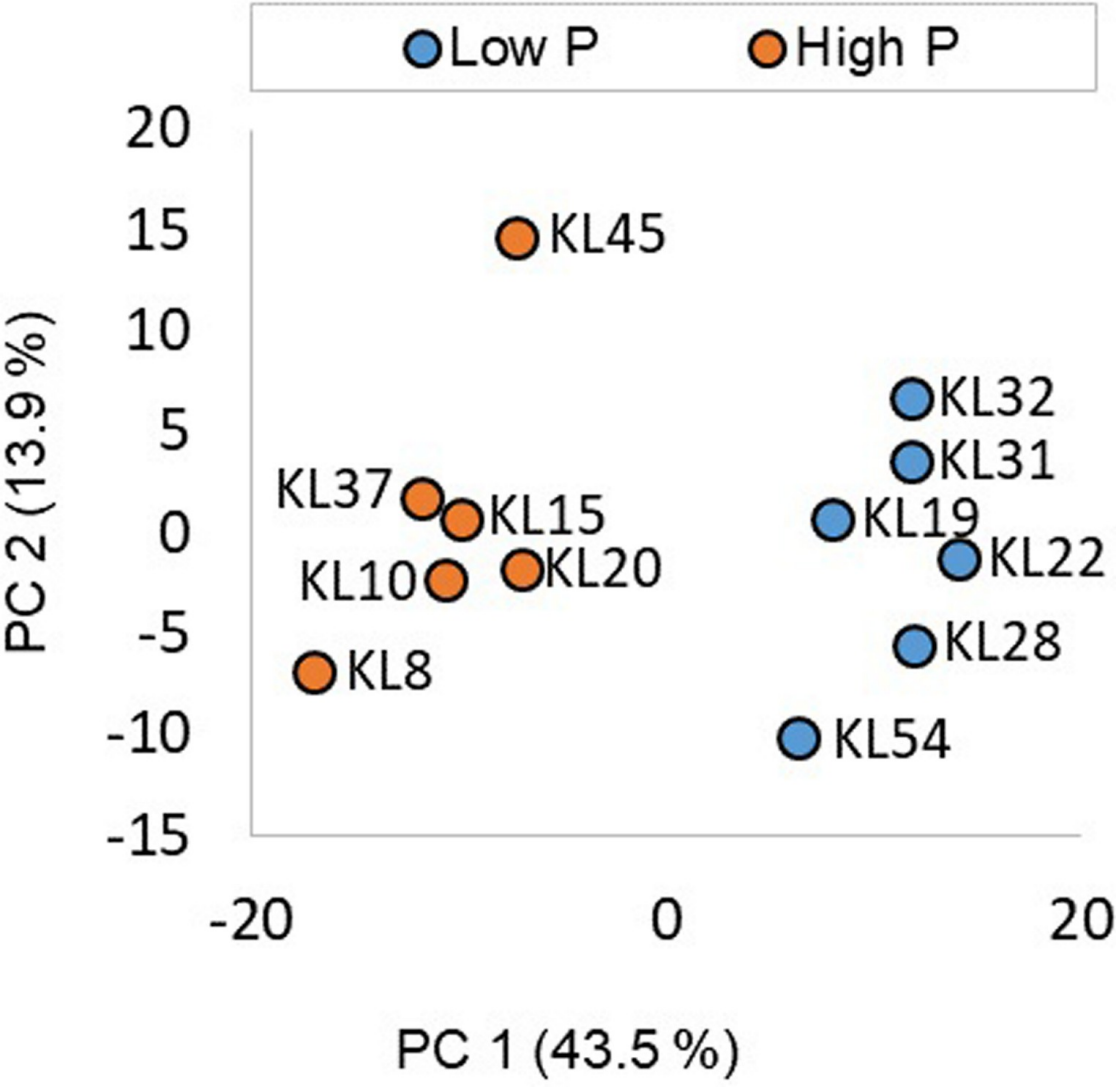
Principal component analysis of the total lipidome of samples with high phosphorous content (orange) versus samples with low phosphorous content (blue). Data was normalized by sum, log transformed and mean centered. PC1 explained 43.5% of the variation, and PC2 explained 13.9% of the variation.

In total, 600 unique molecular lipid species across 36 lipid classes (S5 Table) were tentatively annotated using exact mass and MS/MS information by LipidMatch Flow[21,22]. Acronyms of the lipid classes covered in this manuscript are defined in S5 Table. The total lipidome of the samples grouped based on foliar P content without any exceptions (Fig 1).

Most classes of phospholipids were significantly down-regulated in P-depleted leaves including PC and PE, which have been reported as the most abundant phospholipids in three species of seagrasses[23], whereas diacylglyceryltrimethylhomoserine (DGTS), triglycerides (TG), galactolipid digalactosyldiacylglycerol (DGDG), certain species of glucuronosyldiacylglycerols (GlcADG), and certain species of sulfoquinovosyl diacylglycerol (SQDG) were significantly upregulated (Table 2, S1 Fig, S4 and S5 Tables) and presumably replace phospholipids in the membranes.

**Table 2:** Selected up- and down-regulated lipid classes based on a fisher's exact test.

Lipid class	Fisher's P-value	Up or down-regulated*	Fold Change (low/high P)	T-Test P-value**
**PE**	0.00002	Down	0.6	0.006
**PC**	0.00010	Down	0.5	0.003
**PA**	0.00035	Down	0.5	0.017
DG	0.00053	Down	0.5	0.003
**LPC**	0.00069	Down	0.6	0.017
**OxPG**	0.00080	Down	0.5	0.011
Cer-NS	0.00443	Down	0.6	0.004
DGTS	< 0.00001	Up	1.8	0.014
TG	< 0.00001	Up	1.1	0.176
DGDG	0.00727	Up	1.2	0.204
OxTG	0.03723	Up	1.4	0.001

Lipids in bold contain a phosphate group. See S1 Fig, and S4 and S5 Tables for additional details.

*Up-regulated and down-regulated mean that the lipids were higher or lower, respectively, in leaves with low phosphorus content versus leaves with high phosphorus content based on fisher's exact test.

**T-test (unequal variance, two-sided) of total intensities for sum of lipid class

**Fig 2:**
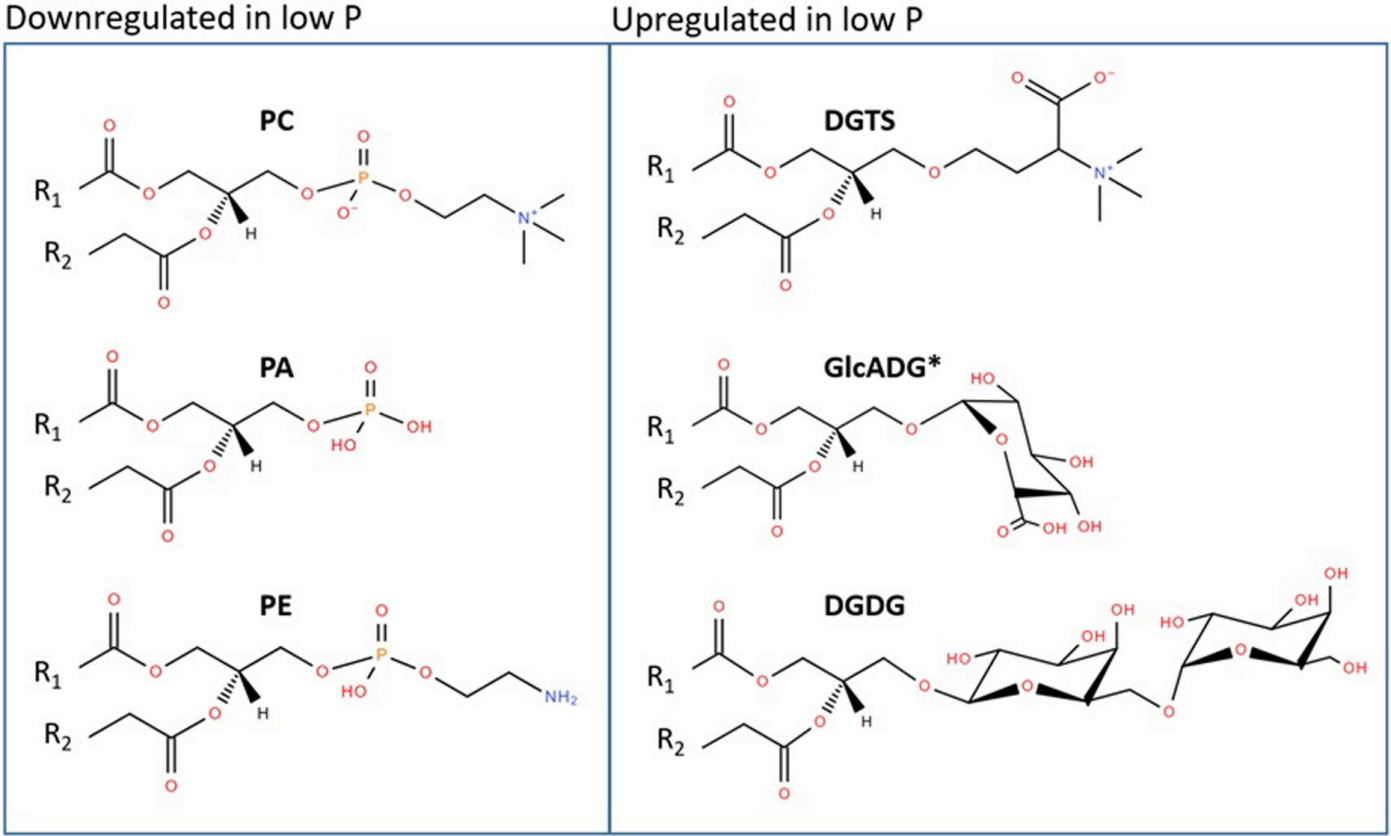
Examples of downregulated phospholipids that are key lipid species within the cell membrane, and significantly upregulated lipids that do not contain phosphorus. Lipid acronyms are defined as follows: phosphatidylcholine (PC), phosphatidic acid (PA), phosphatidylethanolamine (PE), diacylglyceryltrimethylhomoserine (DGTS), glucuronosyldiacylglycerols (GlcADG), and galactolipid digalactosyldiacylglycerol (DGDG). *for GlcADG only a few molecular lipid species were significantly upregulated.

Structures of certain upregulated and downregulated lipids are shown in Fig 2. It is interesting to note that total DGTS had the greatest fold change increase in low P, as compared to other non-phosphorus containing membrane lipids, suggesting partial replacement of the dominant PC membrane lipid. Substitution of phospholipids by non-phosphate containing lipids was first reported in *Proteobacteria*[24], where glycolipids replaced a large part of phospholipids in *Pseudomonas diminuta* so dramatically that in P-limited cultures, phosphate lipids were barely detectable (< 0.3% of total polar lipids). Since this landmark discovery, several lipid classes have been identified in a variety of diverse organisms to be involved in membrane lipid reconstructions during P starvation: SQDG was detected to substitute for phospholipids and thus to reduce P needs in *Arabidopsis* and certain species of picocyanobacteria[25–27]. Similar modifications of membrane lipids, but with DGDG replacing phospholipids, have been reported in oat[28] as well as in seven other species of monocots and dicots[29]. DGTS (a P-free betaine-lipid analog of PC) has been reported to replace PC in fungi[30]. So far, the only study revealing that membrane re-modeling is an important adaptation to low P concentrations in environmental mixed communities was reported for phytoplankton communities in the Sargasso Sea[27].

Using LC-tandem MS and LipidMatch Flow software[21,22], we were able to identify that not all molecular species in a given lipid class showed the same trend and thus the data in Table 2 only shows a simplistic overview of the changes in lipid composition. Under P-depleted conditions, the most significantly upregulated lipid species in *Thalassia* in terms of fold-change were actually GlcADG (S1 Fig, S5 Table), which were only recently discovered in the context of P starvation in *Arabidopsis*[31]. Specifically GlcADG(16:0_18:2), fold change of 21, GlcADG(16:0_16:0), fold change of 7, and GlcADG (18:0_18:2), fold change of 7, were significantly higher under P-deplete conditions compared to high P (S2 Fig). Interestingly, of the twelve GlcADG molecular species that were identified, only four were significantly upregulated (Hochberg corrected p-value < 0.05) and only the three listed above had fold changes above 2 (S5 Table). S2 Fig shows examples of three GlcADG species identified by both MS-DIAL and LipidMatch, which had greatly differing fold changes. This impressively illustrates the use of MS and the urgent need for the identification of single molecular lipid species over other techniques that only analyze lipid classes (e.g. 2-D TLC), and explains why GlcADGs are not included in Table 2, which only shows average changes in lipid classes.

Similar to GlcADGs, TGs were highly upregulated in P-deplete *Thalassia* leaves. TGs were also upregulated in nitrogen studies in the alga *Chlamydomonas reinhardtii*[32]. In general, in starvation conditions, membrane phospholipids are expected to decrease due to a shift towards TG synthesis[33] as well as due to replacement by DGTS and DGDG[28]. We found that a significant number of DGDG species increased in P-deficient seagrass leaves (S4 and S5 Tables). Other lipids, which were downregulated under P-deplete conditions, were diglycerides (DG) and ceramides (Cer-NS), (Table 2, S4 and S5 Tables). DGs are involved in DGTS, DGDG, and TG synthesis, all of which were upregulated in P-deficient *Thalassia* leaves. Still, more research is needed to understand the downregulation of DG and Cer-NS in P-deficient seagrass plants.

While the majority of the 32 SQDG species that were identified had fold changes greater than one (indicating upregulation; 27/32), only two were found to be significantly upregulated (Hochberg corrected p-value < 0.05), namely SQDG (16:0_18:4) and SQDG (40:11) (S5 Table). Therefore, according to our study, SQDG had only minor to no upregulation in concentration compared to TG, DGDG, DGTS, and certain GlcADG species and does not seem to play a major role in remodeling of foliar membrane lipids under different P concentrations. While we cannot completely exclude that some of the 600 detected lipids originate from epiphytes or microbial (endo)symbionts that were not completely removed by our washing steps, we are certain that the decrease in P-containing lipids reflect actual changes in the seagrass lipidome as phosphatidylcholine (PC), phosphatidic acid (PA), and phosphatidylethanolamine (PE) have previously been reported to be the main P-lipids in seagrasses.

## Conclusions

In conclusion, we present evidence of a key cellular mechanism employed by a widely distributed marine plant to thrive in nutrient-poor, oligotrophic conditions. These results not only explain the cellular mechanisms driving variability in turtlegrass P content, but also may potentially explain broader shifts in leaf structure or morphology under P-limitation, as membrane fluidity may be heavily influenced by lipid re-modelling. Understanding the biology of seagrasses and their adaptation to changing nutrient concentrations can help in conservation efforts. The lipid composition of seagrasses could be used as a biomarker to identify long-term nutrient limitation, which might not be detectable from periodic monitoring of nutrient concentrations in the surrounding waters.

## Supporting information

S1 Fig Volcano plot (low phosphorus versus high phosphorus) colored by lipid class. (DOCX)

S2 Fig Box pots of GlcADG identified in negative polarity in both MS-DIAL and LipidMatch (raw data prior to normalization). Boxplots show that the degree/occurrence of upregulation was quite different depending on the GlcADG fatty acyl species. In **bold** is the fold change (low / high P). A) GlcADG(16:0_16:0) [M-H]^−^ (**7**), B) GlcADG(16:0_18:1) [M-H]^−^ (**2**), C) GlcADG(16:0_18:2) [M-H]^−^ (**20**).
(DOCX)

S1 Table Reverse phase liquid chromatography mobile phase gradient. (DOCX)

S2 Table Heated electrospray (HESI) source parameters. (DOCX)

S3 Table Q-Exactive mass spectrometers scan parameters. (DOCX)

S4 Table Upregulated, downregulated, and unchanged lipid classes based on a fisher's exact test (see methods for details) and t-test based on the sum of species total intensities for each lipid class. Based on a fisher's exact test: upregulated means a significant number of lipids in a given class were higher in P deficient substrates, while downregulated means that a significant number of lipids in a given class were lower in P deficient substrates as compared to controls. For total intensity and t-tests, upregulated or downregulated mean the total intensity of that lipid class was higher or lower, respectively, in low P versus high P leaves. Bold p-values are considered significant, while highlighted red values are highly significant. Acronyms are defined in S5 Table.
(DOCX)

S5 Table Table of all annotated lipids (confirmed by LipidMatch Flow using MS/MS rule based annotation) with corresponding peak areas, fold changes, and p-values across groups as well as a list of acronyms of lipids. (XLSX)
