# Supplementary figures and images for: Re-modeling of foliar membrane lipids in a seagrass allows for growth in phosphorus-deplete conditions
Source: PLoS One. 2019 Nov 27;14(11):e0218690. doi: 10.1371/journal.pone.0218690 (PMC6880972; doi:10.1371/journal.pone.0218690)

**S1 Fig. Volcano plot (low phosphorus versus high phosphorus) colored by lipid class.**


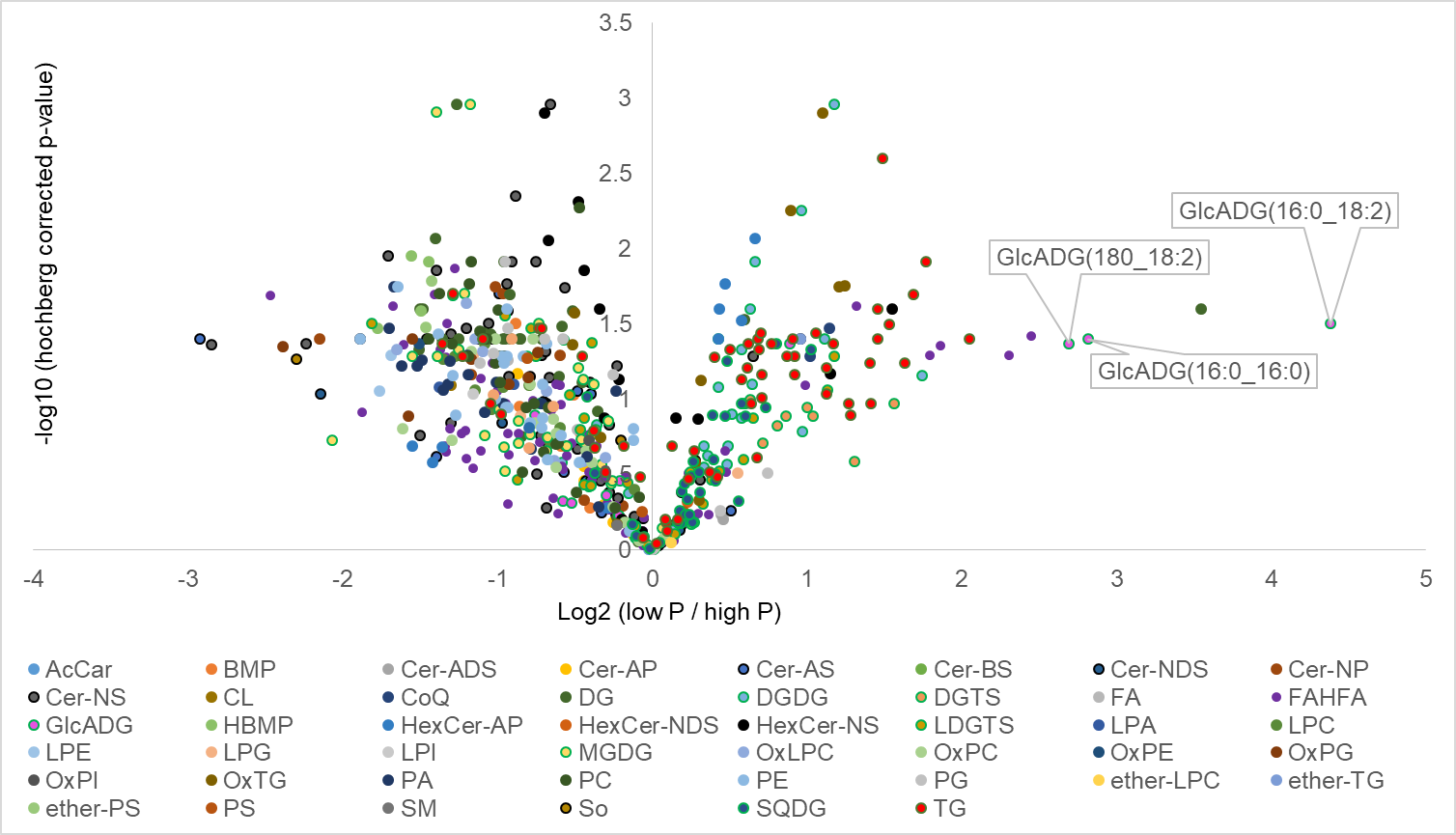

Supplement: S1 Fig — (DOCX) [file pone.0218690.s001.docx]
