# Supplementary material for: Re-modeling of foliar membrane lipids in a seagrass allows for growth in phosphorus-deplete conditions
Source: PLoS One. 2019 Nov 27;14(11):e0218690. doi: 10.1371/journal.pone.0218690 (PMC6880972; doi:10.1371/journal.pone.0218690)

**S2 Fig. Box pots of GlcADG identified in negative polarity in both MS-DIAL and LipidMatch (raw data prior to normalization).** Boxplots show that the degree/occurrence of upregulation was quite different depending on the GlcADG fatty acyl species. In **bold** is the fold change (low / high P). A) GlcADG(16:0_16:0) [M-H]^-^ (**7**), B) GlcADG(16:0_18:1) [M-H]^-^ (**2**), C) GlcADG(16:0_18:2) [M-H]^-^

(**20**).


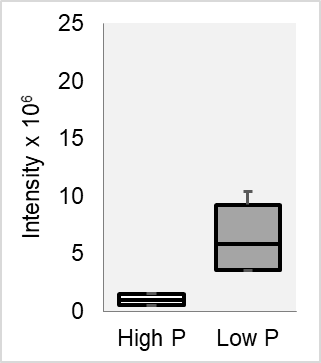

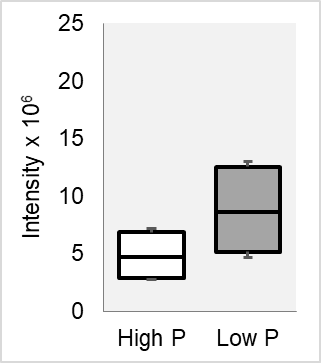

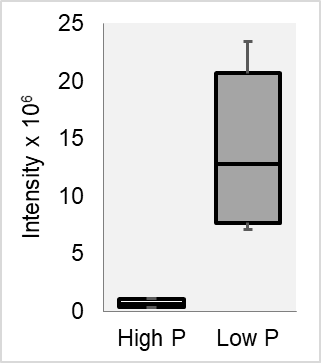

Supplement: S2 Fig — Boxplots show that the degree/occurrence of upregulation was quite different depending on the GlcADG fatty acyl species. In bold is the fold change (low / high P). A) GlcADG(16:0_16:0) [M-H]− (7), B) GlcADG(16:0_18:1) [M-H]− (2), C) GlcADG(16:0_18:2) [M-H]− (20). (DOCX) [file pone.0218690.s002.docx]
