# Supplementary material for: Re-modeling of foliar membrane lipids in a seagrass allows for growth in phosphorus-deplete conditions
Source: PLoS One. 2019 Nov 27;14(11):e0218690. doi: 10.1371/journal.pone.0218690 (PMC6880972; doi:10.1371/journal.pone.0218690)
Supplement: S1 Table — (DOCX) [file pone.0218690.s003.docx]

| **S1 Table. Reverse phase liquid chromatography mobile phase gradient.** | |
| --- | --- |
| **Time (min)** | **%B** |
| 0.00 | 20 |
| 1.00 | 20 |
| 3.00 | 30 |
| 4.00 | 45 |
| 6.00 | 60 |
| 8.00 | 65 |
| 10.00 | 65 |
| 15.00 | 90 |
| 17.00 | 98 |
| 18.00 | 98 |
| 19.00 | 20 |
| 23.00 | 20 |
