# Supplementary material for: Re-modeling of foliar membrane lipids in a seagrass allows for growth in phosphorus-deplete conditions
Source: PLoS One. 2019 Nov 27;14(11):e0218690. doi: 10.1371/journal.pone.0218690 (PMC6880972; doi:10.1371/journal.pone.0218690)
Supplement: S2 Table — (DOCX) [file pone.0218690.s004.docx]

| **S2 Table. Heated electrospray (HESI) source parameters.** | |
| --- | --- |
| Sheath Gas Flow Rate | 30 |
| Aux Gas Flow Rate | 5 |
| Sweep Gas Flow Rate | 1 |
| Spray Voltage | 3.50 |
| Spray Current | (Blank) |
| Capillary Temp | 300 |
| S-Lens RF Level | 35.0 |
| Heater Temp | 300 |
