# Supplementary material for: Re-modeling of foliar membrane lipids in a seagrass allows for growth in phosphorus-deplete conditions
Source: PLoS One. 2019 Nov 27;14(11):e0218690. doi: 10.1371/journal.pone.0218690 (PMC6880972; doi:10.1371/journal.pone.0218690)
Supplement: S3 Table — (DOCX) [file pone.0218690.s005.docx]

| **S3 Table. Q-Exactive mass spectrometers scan parameters.** | |
| --- | --- |
| **Full Scan Parameters:**  Scan Range | 200-1200 |
| Resolution | 70,000 |
| Microscans | 1 |
| AGC target | 3 x 10^6^ |
| Max Injection time | 100 ms |
| **ddMS^2^ parameters:**  Scan Range | 50-1200 |
| Resolution | 35,000 |
| Microscans | 1 |
| AGC target | 5 x 10^6^ |
| Max Injection time  HCD NCE | 175 ms  20, 25, 30 |
| **AIF parameters:**  Scan Range | 146.7-1080 |
| Resolution | 70,000 |
| Microscans | 1 |
| AGC target | 5 x 10^6^ |
| Max Injection time  HCD NCE | 256 ms  20, 25, 30 |
