# Supplementary material for: Re-modeling of foliar membrane lipids in a seagrass allows for growth in phosphorus-deplete conditions
Source: PLoS One. 2019 Nov 27;14(11):e0218690. doi: 10.1371/journal.pone.0218690 (PMC6880972; doi:10.1371/journal.pone.0218690)
Supplement: S4 Table — Based on a fisher's exact test: upregulated means a significant number of lipids in a given class were higher in P deficient substrates, while downregulated means that a significant number of lipids in a given class were lower in P deficient substrates as compared to controls. For total intensity and t-tests, upregulated or downregulated mean the total intensity of that lipid class was higher or lower, respectively, in low P versus high P leaves. Bold p-values are considered significant, while highlighted red values are highly significant. Acronyms are defined in S5 Table. (DOCX) [file pone.0218690.s006.docx]

**S4 Table. Upregulated, downregulated, and unchanged lipid classes based on a fisher's exact test (see methods for details) and t-test based on the sum of species total intensities for each lipid class.** Based on a fisher's exact test: upregulated means a significant number of lipids in a given class were higher in P deficient substrates, while downregulated means that a significant number of lipids in a given class were lower in P deficient substrates as compared to controls. For total intensity and t-tests, upregulated or downregulated mean the total intensity of that lipid class was higher or lower, respectively, in low P versus high P leaves. Bold p-values are considered significant, while highlighted red values are highly significant.

*Upregulated and downregulated mean that the lipids were higher or lower, respectively, in low P versus control samples based on a fisher's exact test.

**p-value is based on a student t-test of total intensity
